# Open versus minimally invasive total gastrectomy after neoadjuvant chemotherapy: results of a European randomized trial

**DOI:** 10.1007/s10120-020-01109-w

**Published:** 2020-07-31

**Authors:** Nicole van der Wielen, Jennifer Straatman, Freek Daams, Riccardo Rosati, Paolo Parise, Jürgen Weitz, Christoph Reissfelder, Ismael Diez del Val, Carlos Loureiro, Purificación Parada-González, Elena Pintos-Martínez, Francisco Mateo Vallejo, Carlos Medina Achirica, Andrés Sánchez-Pernaute, Adriana Ruano Campos, Luigi Bonavina, Emanuele L. G. Asti, Alfredo Alonso Poza, Carlos Gilsanz, Magnus Nilsson, Mats Lindblad, Suzanne S. Gisbertz, Mark I. van Berge Henegouwen, Uberto Fumagalli Romario, Stefano De Pascale, Khurshid Akhtar, H. Jaap Bonjer, Miguel A. Cuesta, Donald L. van der Peet

**Affiliations:** 1 Department of Gastro-Intestinal Surgery, Amsterdam University Medical Center, Location VU University, De Boelelaan 1117, ZH 7F020, 1081 HV Amsterdam, The Netherlands; 2 Department of Clinical Epidemiology, Amsterdam University Medical Center, Amsterdam, The Netherlands; 3 Department of Surgery, San Raffaele Hospital, Milan, Italy; 4 Department of Visceral-, Thoracic and Vascular Surgery, University Hospital Carl Gustav Carus, Dresden, Germany; 5 Department of Surgery, Universitätsmedizin Mannheim, Medical Faculty Mannheim, Heidelberg University, Heidelberg, Germany; 6 Department of Surgery, Hospital Universitario de Basurto, Bilbao, Spain; 7 Department of Surgery, Hospital Clínico Universitario de Santiago de Compostela, Santiago de Compostela, Spain; 8 Department of Surgery, Hospital de Jerez, Jerez de la Frontera, Spain; 9 Department of Surgery, Hospital Clínico San Carlos, Madrid, Spain; 10 Department of Surgery, IRCCS Policlinico San Donato, Milan, Italy; 11 Department of Surgery, Hospital del Sureste, Madrid, Spain; 12 Department of Surgery, Karolinska University Hospital, Stockholm, Sweden; 13 Department of Gastro-intestinal Surgery, Amsterdam University Medical Center Location AMC, Amsterdam, The Netherlands; 14 Department of Surgery, ASST Spedali Civili, Brescia, Italy; 15 Department of Surgery, Salford Royal NHS Foundation Trust, Manchester, UK

**Keywords:** Minimally invasive total gastrectomy, Neoadjuvant chemotherapy

## Abstract

**Background:**

Surgical resection with adequate lymphadenectomy is regarded the only curative option for gastric cancer. Regarding minimally invasive techniques, mainly Asian studies showed comparable oncological and short-term postoperative outcomes. The incidence of gastric cancer is lower in the Western population and patients often present with more advanced stages of disease. Therefore, the reproducibility of these Asian results in the Western population remains to be investigated.

**Methods:**

A randomized trial was performed in thirteen hospitals in Europe. Patients with an indication for total gastrectomy who received neoadjuvant chemotherapy were eligible for inclusion and randomized between open total gastrectomy (OTG) or minimally invasive total gastrectomy (MITG). Primary outcome was oncological safety, measured as the number of resected lymph nodes and radicality. Secondary outcomes were postoperative complications, recovery and 1-year survival.

**Results:**

Between January 2015 and June 2018, 96 patients were included in this trial. Forty-nine patients were randomized to OTG and 47 to MITG. The mean number of resected lymph nodes was 43.4 ± 17.3 in OTG and 41.7 ± 16.1 in MITG (*p* = 0.612). Forty-eight patients in the OTG group had a R0 resection and 44 patients in the MITG group (*p* = 0.617). One-year survival was 90.4% in OTG and 85.5% in MITG (*p* = 0.701). No significant differences were found regarding postoperative complications and recovery.

**Conclusion:**

These findings provide evidence that MITG after neoadjuvant therapy is not inferior regarding oncological quality of resection in comparison to OTG in Western patients with resectable gastric cancer. In addition, no differences in postoperative complications and recovery were seen.

## Introduction

Gastric cancer remains one of the most common cancers worldwide, but its incidence varies widely. A high incidence is observed in East-Asia with an incidence of up to 60 patients per 100.000 inhabitants in Korea, whereas the incidence in Northwestern Europe and North America is low, with an incidence of around 5.8 per 100.000 [1]. Based on the results of the MAGIC trail and in accordance with the consensus guidelines of the European Society for Medical Oncology (ESMO), radical gastrectomy with free margins and an adequate lymphadenectomy, and if indicated along with perioperative neoadjuvant chemotherapy, is the standard of care in patients with advanced gastric cancer [2, 3].

With the introduction of minimally invasive techniques in the treatment of gastric cancer, several large Asian studies show beneficial short-term outcomes and comparable long-term outcomes in the treatment of gastric cancer [4–6]. It should be noted that these results are obtained in centers with a high volume of gastric cancer patients in earlier stages of disease due to the availability of screening programs [7, 8]. Not only is the incidence of gastric cancer lower in the West, but patients present themselves with more advanced stages of disease, often localized more proximally in the stomach [1, 9]. Most studies regarding minimally invasive techniques in the treatment of gastric cancer have focused on partial gastrectomy. Minimally invasive total gastrectomy (MITG) remains a challenge due to oncological and technical aspects.

The potential advantages; such as reduced operative trauma may lead to reduced postoperative complications and improved postoperative recovery whilst preserving oncological outcomes. These potential benefits have led to widespread adoption of minimally invasive gastrectomy. The results of the MAGIC trial showed that perioperative chemotherapy reduced tumor size and increased progression-free and overall survival in gastric cancer patients. The results of perioperative FLOT versus perioperative ECF in advanced gastric cancer showed a better overall survival in the FLOT group. Based on these results neoadjuvant chemotherapy is standard of care in the treatment of advanced gastric cancer in the West, as advised by the European guidelines [10, 11]. To date no randomized clinical trial has investigated the benefits of MITG, following neoadjuvant therapy, in the Western population. One trial assessed open total gastrectomy (OTG) and MITG, before the implementation of neoadjuvant therapy [12].

We hypothesize that a novel surgical technique such as MITG should at least be non-inferior with regard to oncological quality of the resection, with similar or improved postoperative outcomes and survival. Oncological quality of the resection was measured as the number of retrieved lymph nodes in the corresponding stations and radicality (negative circumferential resection margin) of the procedure.

## Methods

### Study design

A non-inferiority, multicenter, international, randomized trial between January 2015 and June 2018 was performed in 13 hospitals in six European countries. Hospitals in the Netherlands, Sweden, Spain, Italy, Germany and the United Kingdom participated in this trial. The medical ethics board of all participating hospitals approved the trial protocol prior to inclusion of patients.

Eligible patients had resectable adenocarcinoma of the stomach and were treated with neoadjuvant chemotherapy, and based on location of the tumor, total gastrectomy was indicated. Patients had to be above 18 years of age and an American Society of Anesthesiologist classification of 3 or lower. Patients with early gastric cancer suitable for local treatment, distal gastric cancer suitable for distal gastrectomy, gastro-esophageal junction tumors, previous surgery of the stomach and previous or co-existing other malignancies were excluded [13].

For quality assurance, the principal investigator visited all centers interested in trial participation for a site initiation. With a low incidence of gastric cancer in the West, all gastrectomies are performed in specialized hospitals to maintain quality of care for these patients. To prevent surgeon bias, both procedures were done by surgeons experienced in open and minimally invasive gastrectomy. Surgeons eligible for participation had to have experience with at least 20 laparoscopic gastrectomies, consisting of at least ten total gastrectomies. Because this study reflected current practice, no additional quality measures were deemed necessary.

Diagnosis and staging were established before neoadjuvant chemotherapy by esophagogastroscopy, with biopsies and CT scan. Additional diagnostics such as PET-CT, MRI, ultrasound or diagnostic laparoscopy could be performed if deemed necessary. A diagnostic laparoscopy before the start of neoadjuvant chemotherapy was advised if there was doubt regarding resectability. Surgeons at the outpatient clinic informed eligible patients of the treatment regimen. Written informed consent was obtained from included patients.

### Randomization

A computer-generated randomization tool was used to randomize the patients in a 1:1 ratio to either an open or a minimally invasive procedure. Randomization was stratified per study center and performed in blocks of six. Randomization took place after assessment of the inclusion and exclusion criteria and after signing the informed consent form. Both patient and the surgeon were not blinded for the allocated procedure. In one hospital a coin flip on the operating room was used for randomization of several patients due to an error with the online randomization tool at that time. These patients were added as soon as the tool was working again, to ensure block randomization was still correct. To assure the quality of the primary outcome regarding lymphadenectomy and radicality, the pathologist was blinded for the type of surgery.

### Procedures

All patients received neoadjuvant chemotherapy in accordance with local protocols. An overview of types of neoadjuvant chemotherapy are depicted in Table 1.

**Table 1:** Baseline characteristics

Baseline characteristics						
		Open		MI		*p* value
**Total**		49		47		
**Gender (male, %)**		32	65.3%	28	59.6%	0.674
**Age (years, mean ± SD)**		61.8	10	59.4	12.5	0.298
**Length (cm, mean ± SD)**		169.7	11.1	166.7	8.3	0.141
**Weight (kg, mean ± SD)**		72.7	14.6	73.6	13.6	0.765
**BMI (kg/m**^**3**^**, mean ± SD)**		25.2	4	26.5	4.8	0.139
**Weight loss (Yes, %)**		23	50.0%	18	40.0%	0.402
**ASA classification**						
ASA I		6	12.2%	4	8.5%	0.813*
ASA II		31	63.3%	30	63.8%	
ASA III		12	24.5%	13	27.7%	
**WHO-performance status**						
WHO 0		32	65.3%	29	61.7%	0.582*
WHO 1		13	26.5%	16	34.0%	
WHO 2		4	8.2%	2	4.3%	
**Smoking**		15	30.6%	14	29.8%	0.999
Pack years (median, IQR)		37	(20–60)	20	(10–33)	0.115
**Alcohol (Y/N)**		14	28.6%	14	29.8%	0.999
Units/week (median, IQR)		7	(3–14)	5	(2–12)	0.300
**Medical history**						
Cardiovascular		24	49.0%	17	36.5%	0.223
Gastro-intestinal		14	28.6%	15	31.9%	0.825
Endocrine		10	20.4%	13	27.7%	0.477
**Previous abdominal surgery**		15	31.3%	12	25.5%	0.650
**Medication**		35	71.4%	34	72.3%	0.999
PPI		23	52.3%	20	2.0%	0.670
OAC		2	4.5%	1	2.3%	0.999
IDDM		3	6.8%	2	4.5%	0.999
**Preoperative workup**						
**Gastroscopy location**						
Proximal		14	28.6%	13	27.7%	0.979*
Middle		25	51.0%	25	53.2%	
Distal		10	20.4%	9	19.1%	
**Clinical TNM**						
T1		1	2.0%	2	4.3%	0.730*
T2		8	16.3%	9	19.1%	
T3		36	73.5%	30	63.8%	
T4		4	8.2%	6	12.8%	
N0		17	34.7%	17	36.2%	0.711*
N1		25	51.0%	26	55.3%	
N2		7	14.2%	4	8.5%	
**Neoadjuvant therapy**						
ECC		12	24.5%	9	19.1%	0.690*
ECF		10	20.4%	9	19.1%	
EOX		13	26.5%	13	27.7%	
FOLFOX		0		2	4.3%%	
FLOT		10	20.4%	8	17.0%	
Other		4	8.2%	6	12.8%	

* Additional testing within groups with Bonferroni correction showed no differences between groups

Anaesthesia was similar for patients in open or minimally invasive surgery. All patients received a single, prophylactic gift of antibiotics according to the local protocol in the participating centers 30 min preoperatively.

Gastrectomy was performed 6–8 weeks after completion of neoadjuvant chemotherapy. Access to the abdomen in the OTG group was obtained via a median laparotomy. In the MITG group, the placement and number of trocars were based on surgeon preferences. An overview of number of trocars and site of extraction are depicted in Table 2.

**Table 2:** Operative details

Operative details					
	Open (*n* = 49)		MI (*n* = 47)		*p*-value
**Duration of surgery (median, IQR)**	200	(164–245)	244	(198–293)	**0.005**
**Blood loss, ml (median, IQR)**	200	(100–400)	171	(64–300)	0.454
Transfusion (Y/N)	4	8.2%	2	4.3%	0.678
**Peri-operative complications**					
Bleeding	4	8.3%	5	10.6%	0.774*
Bowel perforation	1	2.1%	0		
Arrythmia	1	2.1%	1	2.1%	
**Drain**	39	83.0%	33	70.2%	0.223
**SMEQ-score (median, IQR)**	12.5	(0–67)	50	(0–78.5)	0.393
Minimally invasive total gastrectomy					
**Number of trocars**					
3 trocars			5	10.6%	
4 trocars			12	25.5%	
5 trocars			27	57.4%	
6 trocars			3	6.4%	
**Extraction site**					
Pfannenstiehl			23	48.9%	
Epigastrio			4	8.5%	
Left pararectal			9	19.1%	
Midline mini laparotomy			11	23.4%	
**Conversion**			3	6.4%	
Open total gastrectomy					
**Length of Incision, cm**	20	(17–32)			

* Additional testing within groups with Bonferroni correction showed no differences between groups

Recommended lymph node dissection consisted of a D2 lymphadenectomy, being lymph node stations 1–7, 8a, 9, 10, 11p and 12a [14]. After extraction of the specimen the surgeon attached numbered tags in correspondence with the number of the lymph node station. The specimen was analysed by the pathologist for radicality, the number of resected lymph nodes with the corresponding lymph node stations and response to neoadjuvant chemotherapy according to Mandard [15]. The pathologists had separate access to the online case report forms to enter their results.

Postoperative treatment was similar in both groups. A nasojejunal tube could be inserted according to local preference. Enteral feeding could be resumed depending on the clinical status of the patient. Patients were encouraged to get out of bed and start walking with the help of a physical therapist or nurse. Follow-up took place 3, 6 and 12 months postoperatively, or sooner if deemed necessary.

### Study outcomes

The number of resected lymph nodes with a D2 lymphadenectomy according to the definition of the Japanese Gastric Cancer Association, alongside radicality, is a marker for the quality of care and, therefore, our primary outcome [14]. A standardized report regarding tumor size, radicality, number of resected lymph nodes, resected lymph node stations and Mandard scores was conducted [15].

Secondary outcomes regarded operative results such as operation duration, blood loss and perioperative complications. Postoperative outcomes regarded morbidity and mortality with postoperative mortality being defined as 30-day mortality or in hospital mortality. Complications were graded according to the Clavien–Dindo classification and all complications per patient were assessed using the comprehensive complication index [16, 17]. Complications requiring invasive intervention of admission to an intensive care unit, as measured by a Clavien–Dindo score of 3A or higher, were deemed major complications.

In addition, postoperative recovery such as time to first intake, time to first flatus and hospital stay was measured. In the overall analysis, textbook outcome was determined for each patient. A textbook outcome was achieved if: a curative resection was performed, with no perioperative complications, a pathological radical resection (R0) with more than 15 lymph nodes resected, no major complications (Clavien–Dindo 3A or higher), no reinterventions, a length of hospital stay less than 21 days, no mortality in the first 30 days and no readmission in the first 30 days [18].

Data regarding 1-year disease free and overall survival was collected.

### Statistical analysis

The sample size was calculated based on two previous studies. The first sample size was based on the Dutch cancer registry of 2013, with a median yield of 19 lymph nodes, for non-inferiority with a power of 90% and one-sided *t* test with a significance level (*α*) of 0.05 and a non-inferiority margin of − 4, a total of 66 patients per group were deemed necessary [19, 20].

The second sample size was based on a large western study regarding patients that underwent gastrectomy for cancer with curative intent and described lymph node yield following neoadjuvant therapy in a Western population [21]. For a non-inferiority test with 90% power, and one-sided *t* test with a significance level (α) of 0.05, a standard deviation of 7.8 and non-inferiority margin of − 5 a total of 48 patients per group were to be included. Although the second sample size was more in fit with the study, we were confident that we could reach our first sample size. Accrual started according to the expected scheme, but accrual was slow. Based on the advice of the statistician and medical ethical review board, the research group decided to reassess the sample size.

The main reason for this slow accrual was a large group of patients that did not meet the inclusion criteria, mainly patients were not deemed fit enough for neoadjuvant chemotherapy.

Statistical analysis was performed using SPSS statistical package, version 22 (IBM software). Open and minimally invasive total gastrectomy were compared with continuous variables described as means and standard deviation for normal distributions and medians and interquartile ranges for non-normal distributions. Analysis was performed as intention to treat. Comparison tests were performed with Student’s *t* test and Mann–Whitney *U* tests as appropriate.

Frequencies were described as number and percentage of total. Comparison was performed with Chi square tests, for variables with multiple categories additional testing within groups was performed with Bonferroni correction. A two-sided *p* value of 0.05 was deemed statistically significant. Correction for possible confounders or effect modifiers was assessed using logistic regression techniques. Kaplan–Meier curves with a Mantel–Cox log rank test were used for survival. Cox regression was used to analyze confounders and effect-modifiers.

## Results

### Patient characteristics

**Fig. 1:**
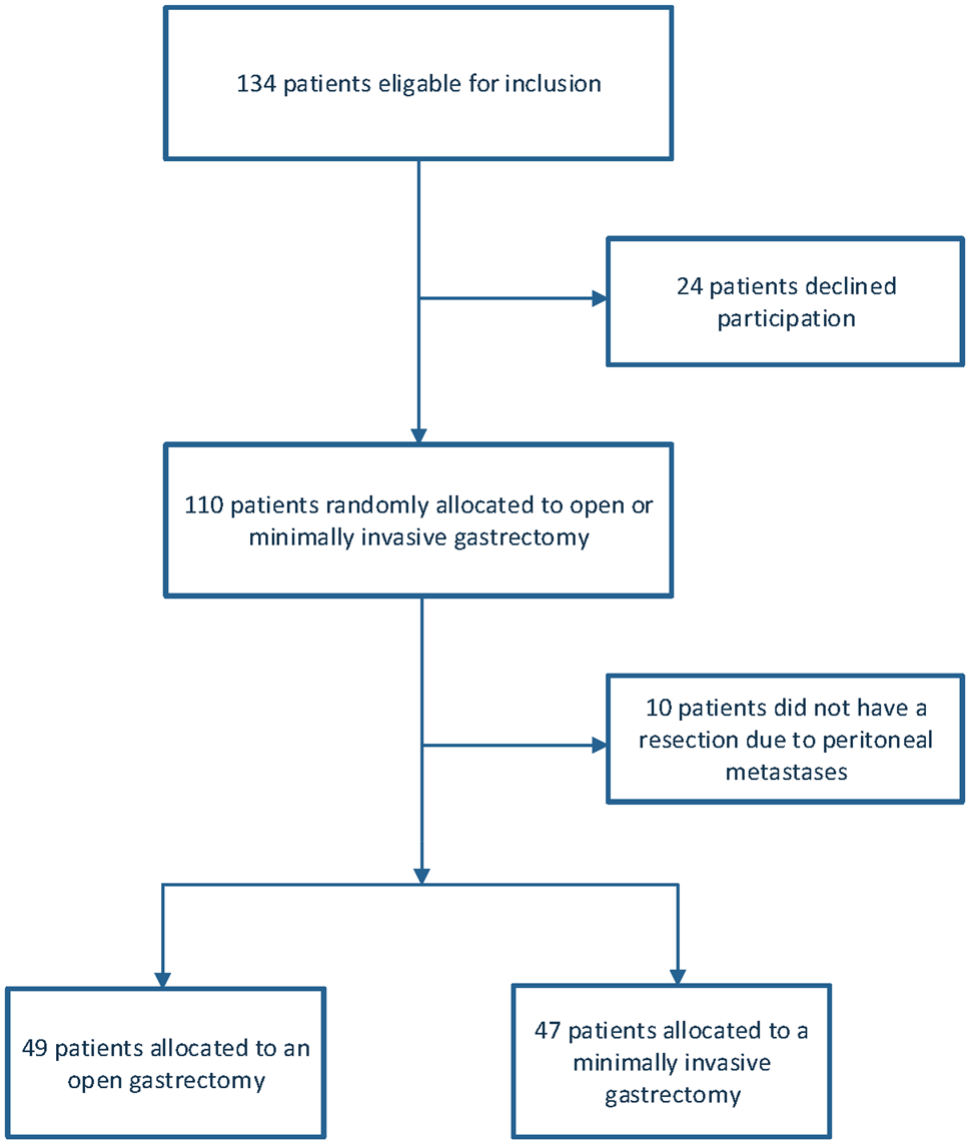
Flowchart of the included patients

A total of 110 patients of 134 eligible patients were included in this trial and randomly assigned to undergo an open or minimally invasive gastrectomy. In ten patients, gastrectomy was not performed due to peritoneal carcinomatosis at the time of surgery. These patients were excluded from further analyses. Four patients retracted from the trial and therefor the data could not be used for analyses, thus resulting in 96 patients. A flow chart of the included patients is depicted in Fig. 1.

The demographic and clinical characteristics of the two groups were similar at baseline. More men (57.6%) than women (42.4%) were included in this trial (*p* = 0.525).

Average age of included patients was 61.8 ± 10 years in the OTG group and 59.4 ± 12.5 years in the MITG group (*p* = 0.298). Preoperative weight and BMI showed no difference, but a large group of patients suffered preoperative weight loss. All patients underwent a gastroscopy and staging CT, at the discretion of the local surgeon additional preoperative diagnostics were performed. A total of eight patients underwent diagnostic laparoscopy to assess resectability prior to starting neoadjuvant chemotherapy.

In the OTG group 14 patients (28.6%) had a proximal tumor, 25 patients (51.0%) a middle tumor and ten patients (20.4%) a distal tumor. In the MITG group 13 patients (27.7%) had a proximal tumor, 25 (53.2%) a middle tumor and nine (19.1%) a distal tumor. There was no significant difference in distribution between both groups (*p* = 0.979). Baseline characteristics and preoperative workup are depicted in Table 1.

### Oncological outcomes

Oncological safety of the procedure was measured as the number of retrieved lymph nodes and radicality of the procedure.

Pathological examination of the resected specimen showed that the numbers of retrieved lymph nodes and completeness of resection (i.e., resection margin (R0)) were similar between both groups. The mean number of resected lymph nodes was 43.4 ± 17.3 in OTG and 41.7 ± 16.1 in MITG (*p* = 0.612), mean difference 1.728 (95% CI − 5.02–8.47). For all lymph node stations data was collected as to whether the surgeon reported the station as resected. Next the pathologist confirmed resection for each tagged station if lymphoid tissue was found in that station. The number of harvested lymph nodes per station were also reported. No differences were observed in resected lymph node stations between both groups. A full D2 lymphadenectomy was performed in 49% of OTG versus 36.2% of MITG (*p* = 0.223). A splenectomy was performed in 12.2% OTG cases, versus 4.3% in MITG cases (*p* = 0.269).

There was no significant difference in radicality. Four patients had positive resection margins, one in the OTG and three in the MITG group. In 89.6% of all patient’s histological report showed adenocarcinoma with 51.0% intestinal type in the OTG group versus 34.0% in the MITG group and 38.8% diffuse type in the OTG group versus 55.3% in the MITG group, this was not significantly different.

No significant differences were observed for textbook outcome. A textbook outcome was reached in 77,6% of patients in the OTG group versus 66% in the MITG group (*p* = 0.258).

An overview of all oncological results is depicted in Tables 3 and 4.

**Table 3:** Pathology

Pathology results					
	Open (*n* = 49)		MI (*n* = 47)		*p*-value
**Pathology type**					
Intestinal type adenocarcinoma	25	51.0%	16	34.0%	0.366*
Diffuse type adenocarcinoma	19	38.8%	26	55.3%	
Carcinoid	1	2.0%	1	2.1%	
Signet cell carcinoma	3	6.1%	1	2.1%	
Other	1	2.0%	3	6.4%	
**ypT-stage**					
T0 (complete regression)	4	8.2%	3	6.4%	
Tis	2	4.1%	1	2.1%	
T1	8	16.3%	6	12.8%	
T2	4	8.2%	5	10.6%	
T3	19	38.8%	17	36.2%	
T4	12	24.5%	15	31.9%	
**ypN-stage**					
N0	23	46.9%	20	42.6%	
N1	13	26.5%	7	14.9%	
N2	8	16.3%	8	17.0%	
N3	5	10.2%	12	25.5%	
**Radical resection**					
Proximal margin	0		3	5%	0.242
Distal margin	1	2.2%	0		0.495
**Lymph nodes**					
**Total LN**	43.4	±17.3	41.7	± 16.1	0.612
Micrometastasis	3.2		4.4		0.514
Macrometastasis	1.4		2.1		0.332
**Station 1**					
Surgeon stated as resected	49	100%	47	100%	NA
LN in station (Y/N)	44	89.8%	45	95.7%	0.549
Average LN yield	3	(2–6)	3	(2–6)	0.934
**Station 2**					
Surgeon stated as resected	49	100%	47	100%	NA
LN in station (Y/N)	44	89.8%	44	93.6%	0.610
Average LN yield	2	(1–4)	2	(1–3)	0.181
**Station 3**					
Surgeon stated as resected	49	100%	47	100%	
LN in station (Y/N)	45	91.8%	47	100.0%	0.387
Average LN yield	5	(3–15)	3	(2–8)	0.019
**Station 4**					
Surgeon stated as resected	49	100%	47	100%	NA
LN in station (Y/N)	46	93.9%	47	100.0%	0.368
Average LN yield	6	(2–11)	5	(2–12)	0.770
**Station 5**					
Surgeon stated as resected	48	98.0%	45	95.7%	0.613
LN in station (Y/N)	45	93.8%	44	97.8%	0.549
Average LN yield	1	(0–3)	1	(0–3)	0.381
**Station 6**					
Surgeon stated as resected	49	100%	47	100%	NA
LN in station (Y/N)	45	91.8%	46	97.9%	0.513
Average LN yield	4	(2–9)	4	(2–7)	0.487
**Station 7**					
Surgeon stated as resected	48	98.0%	46	97.9%	0.999
LN in station (Y/N)	44	91.7%	44	95.7%	0.610
Average LN yield	3.5	(2–7)	3.5	(1–6.5)	0.580
**Station 8**					
Surgeon stated as resected	48	98.0%	45	95.7%	0.613
LN in station (Y/N)	46	95.8%	42	93.3%	0.147
Average LN yield					
**Station 9**					
Surgeon stated as resected	49	100%	44	93.6%	0.113
LN in station (Y/N)	39	79.6%	40	90.9%	0.589
Average LN yield	2	(1–4)	4	(1–6)	0.125
**Station 10**					
Surgeon stated as resected	27	57.4%	23	48.9%	0.683
LN in station (Y/N)	20	74.1%	21	91.3%	0.597
Average LN yield	1.5	(0–4)	1	(0–4)	0.989
**Station 11**					
Surgeon stated as resected	42	89.4%	36	76.6%	0.302
LN in station (Y/N)	35	83.3%	30	83.3%	0.327
Average LN yield	2	(0–3)	1	(0–3)	0.796
**Station 12**					
Surgeon stated as resected	39	83.0%	35	74.5%	0.631
LN in station (Y/N)	28	71.8%	31	88.6%	0.52
Average LN yield	1	(0–2)	2	(0–4)	0.096
**Station 13**					
Surgeon stated as resected	1	2.1%	0		0.999
LN in station (Y/N)	0		0		
Average LN yield	–		–		

* Additional testing within groups with Bonferoni correction showed no differences between groups

** Additional testing within groups revealed there were significantly more N3 patients in the minimally invasive group

**Table 4:** Oncological results

Oncological results						
		Open (*n* = 49)		MI (*n* = 47)		*p* value
**Lymph nodes**						
Total (mean ± SD)		44.3	±16.7	40.7	± 16.3	0.209
Micrometastasis		3.2		4.4		0.514
Macrometastasis		1.4		2.1		0.332
**Radicality**						
R0 resection		48	98.0%	44	93.6%	0.617
Positive proximal margin		0		3	5.0%	0.242
Positive distal margin		1	2.2%	0		0.495
**Mandard classification**^a^						0.934*
1	Complete regression (= fibrosis without detectable tissue of tumor)	6	14.0%	5	11.4%	
2	Fibrosis with scattered tumor cells	7	16.3%	5	11.4%	
3	Fibrosis and tumor cells with preponderance of fibrosis	7	16.3%	9	20.5%	
4	Fibrosis and tumor cells with preponderance of tumor cells	12	27.9%	12	27.3%	
5	Tissue of tumor without changes of regression	11	25.6%	13	29.5%	

* Additional testing within groups with Bonferroni correction showed no differences between groups

^a^Data missing of 9 patients

Regression techniques determined no confounding or effect modification by sex, age, BMI, T-stage and N-stage for these outcomes.

### Operative outcomes

Although duration of surgery was significantly longer in the MITG group, with a median length of 244 min (IQR 198–293 min) in comparison to 200 min (IQR 164–245 min) in the OTG group (*p* = 0.005), blood loss was similar in both groups, with a median of 200 ml in the OTG and 171 ml in the MITG group (*p* = 0.454). In three patients the minimally invasive procedure was converted to an open procedure. Reasons for conversions were technical difficulty due to tumor infiltration, adhesions and perioperative hemodynamic instability.

In nine patients perioperative bleeding was reported, five in the MITG and four in the OTG group and one hospital reported an iatrogenic perforation of the colon which was directly repaired.

Operative details are depicted in Table 2.

### Postoperative outcomes

Regarding postoperative recovery, outcomes were similar in both groups. Mean hospital stay was 8 days (IQR 7–11 days) in both groups if no complications occurred (*p* = 0.338). There was no significant difference in postoperative complications between OTG and MITG, with a total of 42.9% in OTG and 34.0% in MITG (*p* = 0.408). No differences were observed between OTG and MITG for major complications, being Clavien–Dindo grade 3 or higher (*p* = 0.377). Closer assessment of complications like postoperative bleeding, abscesses, anastomotic leakages (five in the OTG group, 10.2% and four in the MITG, 8.5%), and wound dehiscence, one in every group, were not statistically different between OTG and MITG. Both circular stapled (54,2%) and linear stapled (45,8%) techniques were applied equally in OTG and MITG for the formation of the esophagojejunostomy (*p* = 0.546). No differences were observed in anastomotic leak rates between the two techniques (*p* = 0.501). In the OTG group two patients died after anastomotic leakage due to multi organ failure and sepsis, two other patients had a reoperation, and one was treated with antibiotics only. In the MITG group one patient had a reoperation, one had percutaneous drainage and two were treated with antibiotics. Median Comprehensive Complication Index was not significantly different between both groups with a mean of 20.9 (IQR 20.9–31.56) in the OTG and 26.22 (IQR 20.9–33.7) in the MITG group (*p* = 0.242).

The time to first intake was 5 days in both groups, as was the time to first passage of stool. Postoperative results are depicted in Table 5.

**Table 5:** Postoperative results

Postoperative results					
	Open (*n* = 49)		MI (*n* = 47)		*p*-value
**Complications**	21	42.9%	16	34.0%	0.408
**Clavien-Dindo**					
CD 1	3	6.1%	1	2.1%	0.377*
CD 2	12	24.5%	7	14.9%	
CD 3A	1	2.0%	3	6.4%	
CD 3B	2	4.1%	2	4.3%	
CD 4	1	2.0%	3	6.4%	
CD 5	2	4.1%	0	–	
Anastomotic Leak	5	10.2%	4	8.5%	0.527
Abscess	1	2.0%	1	2.1%	0.742
Perforation	1	2.0%	1	2.1%	0.742
Wound infection	1	2.0%	1	2.1%	0.742
Fascial dehiscence	0		0		
Pneumonia	3	6.1%	4	8.5%	0.712
Urinary tract infection	1	2.0%	0		0.510
Bleeding	3	6.1%	0		0.494
Cardial	1	2.0%	1	2.1%	0.742
Thrombo-embolic	2	4.1%	1	2.3%	0.999
Other	8		11		
Technique for anastomosis**					0.546
Circular stapled	25	49%	27	57%	
Anastomotic leak	3		3		
Linear stapled with V-loc	24	51%	20	43%	
Anastomotic leak	2		1		
**Postoperative recovery (days)**					
Hospital stay					
Uncomplicated	8	(7–11)	8	(7–9)	0.338
Complicated	11	(9–15)	10	(8–17)	0.452
First intake	4	(3–6)	4	(3–6)	0.641
First stool	5	(4–6)	5	(4–6)	0.707

* Additional testing within groups with Bonferroni correction showed no differences between group

^a^No differences were observed in anastomotic leak rates between the two techniques (*p* = 0.501)

**Fig. 2:**
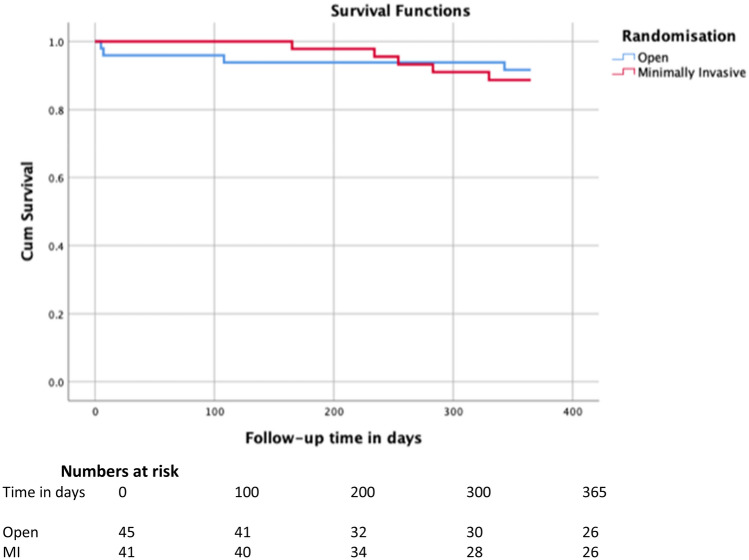
1-year survival

No differences were seen in the 1-year survival between the OTG and MITG group. In the OTG group 90.4% and in the MITG group 85.5% was alive 1 year after surgery (*p* = 0.701). One-year survival curves are depicted in Fig. 2.

## Discussion

In this trial, MITG and OTG after neoadjuvant therapy resulted in similar oncological outcomes; measured by number of retrieved lymph nodes, lymph node stations resected, radicality of the resection and 1-year survival. This is the first European randomized trial evaluating oncological outcomes in MITG and OTG. The short-term results of this trial indicate that MITG is non-inferior to OTG regarding oncological and surgical safety.

In gastric cancer, a radical resection and adequate lymph node dissection has shown to be an important prognostic factor and is a marker for the quality of surgery, and thereby quality of care. According to the ESMO guidelines an adequate lymph node dissection comprises of a D2 dissection with a minimum of 15 lymph nodes [3]. This number represents the minimum for an adequate staging but also for adequate resection. In this trial the mean number of resected lymph nodes was 43.4 ± 17.3 in OTG and 41.7 ± 16.1 in MITG group (*p* = 0.612) and within the range of the predetermined non-inferiority margin. Looking more closely at the resected lymph node stations for an adequate D2 lymphadenectomy no differences were observed between the two groups. It should be noted that the successful D2 lymphadenectomy rate was rather low, which was mainly due to inadequate dissection of station 10. Standard dissection of station 10 is no longer recommended in the latest JGCA guidelines unless the tumor invades the greater curvature [22].

Due to the low incidence of gastric cancer in the West compared to Asia, no screening program exists, and patients often present with advanced disease stages [2]. In this study over 60% in both MITG and OTG presented with cT3-4 stages. Due to the majority of patients presenting with more advanced stages of disease, neoadjuvant therapy is more common in the West compared to Asia.

Alongside a higher rate of comorbidities (i.e., cardiovascular, endocrine, pulmonal diseases, and obesity. Measured overall with the ASA classification) was observed. Although average BMI was higher, patients often suffered preoperative weight loss, indicating a poor nutritional status.

In Northern Europe the incidence of gastric cancer has stabilized. Active eradication of Helicobactor Pylori, and diet changes leading up to ingestion of more fresh products (and less preservatives) have led to a decreased incidence in distal gastric cancers, whereas the incidence for proximal and middle gastric cancers seems stable [1]. Indicating the need for optimal surgical strategies for total gastrectomy in these patients.

Postoperative recovery and postoperative morbidity and mortality was similar in both groups. Previous studies on minimally invasive gastrectomy focused mainly on distal gastrectomy, such as the South Korean KLASS trials [23, 24]. These trials showed that minimally invasive distal gastrectomy is oncologically safe with improved postoperative outcomes such as a lower complication rate, faster recovery and less pain compared to open surgery. The short-term advantages found in these trials with distal gastrectomy have not be found in our study, where there are no differences in short-term outcomes between both approaches.

Concerning total gastrectomy, some meta-analyses have been performed showing that MITG had longer operation times, less operative blood loss, similar rates in lymph node yield with the same completeness of the resection, faster postoperative recovery, less postoperative complications with shorter length of hospital stay and similar mortality rates. Both meta-analyses have emphasized the need for a prospective randomized trial comparing MITG and OTG [25, 26]. In a single arm confirmatory trial the safety of LATG was further emphasized [27].

The outcomes regarding postoperative recovery from our trial might be explained by the multi-disciplinary approach to perioperative care. For example, clinical care pathways ensure better postoperative pain management with dedicated pain teams. Alongside, standardized postoperative patient care protocols ensure optimal perioperative care and allows early diagnosis and treatment of complications.

The South Korean KLASS 03 feasibility study on MITG on patients with stage I proximal gastric cancer has found postoperative morbidity rates of 20.6% with grade III or higher of 9.4% and a mortality of 0.6% [28].

The outcome of our trial reflects the daily practice in the West with less incidence of gastric cancer, where centralization in high volume centers, implementation of minimally invasive gastrectomy, and adoption of new neoadjuvant chemotherapy are important items.

Because strict exclusion criteria were met, accrual of patients was slower than expected.

This was attributed to several factors. As reported earlier a large group of patients were not deemed fit for neoadjuvant chemotherapy and, therefore, not included in this trial. In addition, only patients receiving total gastrectomy could be included to maintain a homogenous study population. This is an important shortcoming in the trial, as the sample size was re-adjusted to a lower sample size to ensure adequate completion of the trial.

The discrepancy between postoperative and preoperative staging was remarkable, with many patients being under staged preoperatively. Ten patients were excluded in the trial due to perioperative diagnosis of peritoneal metastases. Despite thorough preoperative workup in patients with gastric cancer, adequate staging remains a challenge [29].

This trial is the first trial to compare MITG and OTG in a Western population, taking into account neoadjuvant treatment. Long-term outcomes concerning survival and disease-free survival will be collected to evaluate long-term results. However, it should be noted that this study was not powered on disease free and overall survival. In addition, future research should also assess quality of life in patients with advanced gastric cancer.

## Conclusion

The results of this trial provide evidence of non-inferiority regarding quality of the oncological resection in MITG compared to OTG in the treatment of advanced gastric cancer. This indicates that MITG is safe from an oncological point of view. Postoperative complications and recovery are similar in OTG and MITG. Awaiting the results of quality of life and 3-year overall survival, currently the type of approach is at the discretion of the surgical team and may depend on patient factors and patient’s choice.
